# Supplementary material for: Are carbon emissions trading and green financial instruments synergistic? -Comprehensive quantitative research based on content analysis
Source: PLoS One. 2024 Mar 7;19(3):e0298601. doi: 10.1371/journal.pone.0298601 (PMC10919601; doi:10.1371/journal.pone.0298601)
Supplement: S1 Data — (ZIP) [file pone.0298601.s001.zip › Data/地级市Z_F工作报告.zip!/╔┬╬≈/░▓┐╡╩╨/└┤╫╘╣1⁄2╓┌║┼í╛┬φ┐╦╩2╛▌═°í┐_2005.docx]

各位代表：

　　现在，我代表市人民政府，向大会作工作报告，请予审议，并请政协委员和其他列席人员提出意见。

一、2004年的工作回顾

　　2004年，市人民政府在中共安康市委的正确领导下，紧紧依靠全市人民，以党的十六大和十六届三中、四中全会精神为指导，坚持科学发展观，团结奋斗，真抓实干，全面完成了市一届人大六次会议确定的各项任务，经济增长的速度和效益都是近年来最好的一年，实现了经济社会协调发展。

　　——经济发展速度加快。预计实现生产总值117亿元，增长9%。农业获得丰收，农业总产值达到54.2亿元，增长12.8%。粮食总产90.38万吨，增长17%。畜牧业产值14.3亿元，增长15.5%。劳务输出44.7万人，净增13万人。工业总产值33.7亿元，增长23.2%。固定资产投资56.2亿元，增长21.2%。其中，公路建设投资3.64亿元，市政基础设施建设投资2.5亿元。第三产业增加值56.9亿元，增长8.1%。社会消费品零售总额35.8亿元，增长12%。财政总收入10.1亿元，增长23.8%。

　　——药水游三大建设扎实推进。以承办首届“中国秦巴药业投资贸易洽谈会”为契机，着力推进中药产业标准化建设，5个县被国家确定为中药材规范化或现代化基地县。实现药业总产值12.6亿元，增长8%。汉江水资源的开发与保护取得新成绩。喜河水电站实现二期截流，蜀河水电站进点施工，旬阳和白河水电站前期准备进度加快。支流开发新增小水电装机10.14万千瓦。为保护汉江水质，实施了高标准工程造林植竹、退耕还林、湿地保护、自然保护区建设，造林67.9万亩。水土水保治理750平方公里。安康江南污水处理厂、五县区垃圾处理工程加紧建设。关闭5户环境违法企业，停产治理7户。以“瀛湖风光”、“汉江龙舟节”、“岚河漂流”为代表的生态旅游在省内外的知名度日益提高，带动了旅游服务水平的提升。旅游景区基础建设投入2750万元。全市标准客房床位达到1.8万张，新增床位3000张，新增餐饮企业126家。全年接待游客108万人次，实现旅游综合收入3.5亿元。

　　——改革开放迈出新步伐。全市启动国企改制172户，完成58户。平利、白河、岚皋等县全面完成国企改制任务。安康丝织印染厂依法破产、石泉缫丝厂拍卖、民威公司兼并市糖酒副食公司的成功，打破了国企大户改革停滞的局面。随着国有资本的退出，非公有制经济迅速发展，已经成为经济增长的主导力量。党政事业单位非经营性国有资产改革顺利启动，实现了市直单位房地产产权集中管理。顺利完成了市政府机构改革。开展了第二轮行政审批制度改革，精减审批事项335项。启动了粮食流通体制改革。以整治投资环境为重点，在全市开展了“诚信安康”大讨论，严肃查处了一批破坏投资环境的典型案件。充分利用“龙舟节”、“西洽会”、“常博会”、“陕港经贸合作周”等平台，扎实做好招商引资工作，全年共签约项目135个，引进资金42.95亿元，已开工建设104个，招商项目当年完成投资9亿元。

　　——社会事业健康发展。进一步重视了各项社会事业的发展，财政预算内专项建设投入达到1.88亿元，是历年来投资最多、增长最快的一年。安康学院的筹建和职业技术学院的成立，整合了高等教育资源，完善了教育体系。汉阴县通过“普九”验收。改造中小学危房17.5万平方米。高考二本上线比上年增加1093人。科技人员创办领办产业项目，为创新农业科技服务体系、深化农技事业单位改革走出了新路。11个疾控中心基本建成，市传染病医院和9县传染病区项目全面开工。7所县医院和46所乡镇卫生院建设进展顺利，地方病、传染病防治和农村儿童计划免疫工作得到重视。10县区全部被命名为省级计划生育综合服务县。新闻宣传为营造稳定和谐的舆论环境发挥了积极作用。信访工作责任制得到较好落实，案件查结率继续保持全省领先位次。“四五”普法向纵深发展。重大刑事案件破案率居全省前列。精神文明创建深入推进。“一县一品”文化建设各具特色。群众体育活动蓬勃开展。慈善协会、红十字会在扶弱帮困中的作用更为突出。外事、气象、地震、档案、方志、老龄、残疾人和妇女儿童等各项事业取得新进步。金融、保险、铁路、电力、通信、石油、烟草等行业为地方经济发展做出了积极贡献。

　　——群众生活有新的改善。农民人均纯收入达到1652元，净增147元。各级财政支农投入3.05亿元，为农民减免税费8500万元，兑现退耕还林和粮食直补资金7.97亿元。实施了126个扶贫重点村建设，移民搬迁7100人，实现6.8万人脱贫，解决了10.8万农民饮水困难。新增和恢复通公路村 117个，基本实现村村通公路。宁陕等重灾区恢复重建进展良好，灾民生活得到妥善安置。对农村2.4万人实施了最低生活保障。对义务教育阶段贫困学生资助书费杂费209万元。对农村部分计划生育家庭实行了奖励扶助。城镇居民人均可支配收入5942元，净增 317元。新增城镇就业9813人，下岗职工实现再就业6157人。有39364名城市低收入居民领到低保金2536万元。群众呼声较高的一些热点难点问题得到较好解决，改造了安康城区22条背街小巷，在边远村落建成便民桥376座，蔺河库区公路改建工程基本竣工，撤销了汉江一桥收费站。

　　——政府自身建设得到加强。各级政府转变职能的自觉性有了提高，尽量减少对微观经济活动的干预，强化了公共服务职能，调整财政支出结构，扩大对社会保障和公共事务的投入。坚持依法行政，认真学习贯彻《行政许可法》，注重增强政府工作人员依法行政的能力。认真执行人大及其常委会的各项决议、决定，自觉接受人大依法监督、政协民主监督和新闻舆论监督。办结人大代表建议145件、政协委员提案188件。对群众关心的城市改造、政府调价、物业管理及征地拆迁等实行了专家咨询、居民听证和公示制度。加强廉政建设，推进政务公开，加大审计、监察、统计执法监督力度，下力纠正部门和行业不正之风。切实改进作风，通过领导示范工程造林、部门支教助学、抽调干部包项目抓积案、重点工作专项督查督办等一系列措施，有力地推进了落实，使求真务实之风得以弘扬。

　　各位代表，过去的一年，全市人民和各级干部通过自己的辛勤劳动，将改革与发展推向了一个自觉实践科学发展观的新境界，全市上下呈现出难能可贵的团结和谐、奋力向上的新局面。在此，我谨代表市人民政府，向全体人大代表、政协委员和各界人士，向驻安部队和公安武警　　官兵，向为安康发展做出贡献的投资者、建设者和纳税人，表示崇高的敬意和诚挚的感谢！

我们虽然取得了一定的成绩,但绝没有任何理由固步自封。应当清醒地看到，我们的增长是大灾之后低水平基础上的增长，我们的发展是国家加大扶持下的发展，我们的进步与人民群众安居乐业、建设美好生活的热望还有很大差距。我们必须正视工作中存在的问题和困难。一是建设资金匮乏，国有投资下降，中小企业贷款十分困难，招商项目资金到位较差，一批重点项目进展艰难。二是财政收支矛盾尖锐，政府债务居高不下，铺张浪费及攀比之风滋长。三是投资环境仍不宽松。路、电、水等基础设施条件很不适应开放的需要，服务意识不强，办事效率不高，扯皮掣肘屡有发生。法制环境、诚信建设问题较多。四是安全稳定工作的基础不扎实。企业改制、征地拆迁和一些涉及群众具体利益而引发的集体上访和越级上访有所增加。社会稳定和安全生产的薄弱环节较多，特别是紫阳县“12·17”沉船事故、吉河信用社抢劫案，集中暴露了我们内部管理不严、安全防范不力、领导失职失察的问题，教训极为深刻。我们还应看到，经济结构不合理特别是工业薄弱的现状，直接影响到城市化进程和对外开放，成为制约我市经济增长质量和效益的关键环节；农民稳定增收的产业基础不牢，扶贫攻坚任务还很艰巨，城乡就业压力加大；受国企改革滞后和民营资本初始积累不足的影响，非公有制经济整体水平较低，尚未出现突破性发展。这些问题我们要在深入调查研究的基础上，在加快改革与发展的进程中着力加以解决。

二、今年经济社会发展的任务

　　2005年是全面完成“十五”计划、为“十一五”奠基的重要一年。我们要以邓小平理论和“三个代表”重要思想为指导，牢固树立和落实科学发展观，坚持发展第一要务，以结构调整为主线，以药水游三大产业建设为重点，夯实农业基础，突出发展工业，努力扩大投资，积极引导消费，放手发展非公有制经济，推动社会事业进步，不断改善群众生活，加快建设和谐安康。预期目标是：生产总值达到135亿元，增长9.5%，全社会固定资产投资62亿元，增长15%，财政总收入达到11.2亿元，增长12%，城镇居民人均可支配收入净增300元，农民人均纯收入净增120元，城镇登记失业率控制在4%以内，人口自然增长率控制在3.5‰以内。

　　安康正处在加快发展的机遇期。西安—安康、武汉—安康—重庆铁路复线、安康铁路枢纽站和西安—安康—重庆高速公路建设在安康境内投资达260亿元，今年将全线开工。如此高强度的投资，不仅会更加突出安康在西部交通大动脉中的枢纽地位，也必将显著增强安康的发展活力与后劲。我市以药水游为重点的绿色经济发展，得到省委、省政府的充分肯定和大力支持，汉江梯级开发、“南水北调”中线工程、中药现代化和生态旅游的实施，必将有力地推进我市经济结构调整和生态环境建设。国家加大西部大开发的投入和实施公共财政政策，对改善贫困地区生产生活条件将产生积极而深远的影响。全市广大干部群众奋起改变面貌的强烈愿望，成为我们加快经济社会发展的强大力量。我们一定要紧紧抓住机遇，增强加快发展的紧迫感、使命感，努力做好今年各方面的工作。

　　（一）推进农业结构调整，夯实农民增收基础

　　按照“稳粮、强牧、壮特”的思路，调整农业产业结构，突出绿色食品开发，提升劳务经济水平，使农民增收建立在可靠的产业支撑上。

着力突破畜禽产业。以畜牧大乡、专业村和专业大户为重点，把专业化经营与千家万户兴牧致富相结合，猪羊禽并举，扩大饲养量，提高商品率。集中资金，出台政策，加大对饲草生产、良种繁育、疫病防治、圈厕沼配套四大建设的扶持和服务，突出畜牧龙头企业的引进和培育，积极推进畜禽产业化。力争基础种畜增长25%以上，商品畜增长15%以上，畜牧业产值增长13%以上，使其成为农民增收的当家产业。

　　巩固壮大特色产业。树立品牌意识，转变增长方式。在建设GAP药源基地的同时，毫不放松地抓好传统地道中药材开发。整合茶业品牌，提高加工品质，依靠质量和品牌增值提效。突出桑园管理，努力扩大发种，推广实用技术，开展综合利用，提高蚕桑生产整体效益。继续抓好烤烟、蔬菜、干鲜果发展。建立绿色商品基地，扩大无公害生产，开展绿色产品认证，发挥平利绞股蓝、紫阳富硒茶国家原产地域保护的优势，以绿色品牌开拓市场。

　　推进产业化经营。鼓励和支持农村各类专业合作社、行业协会和产业大户扩大规模，增强实力；积极引进境外客商来我市投资兴办农产品生产和加工项目；鼓励产业大户反租倒包，实行专业化经营；支持农技人员创办领办产业项目，推动产业化经营；积极探索财政提供贷款担保、由企业或合作经济组织为农户承贷承还的机制；集中捆绑项目和资金，重点投向加工企业、产业大户和带动力强的合作经济组织，不断培育壮大龙头企业。

　　扩大有组织的劳务输出。坚持面向市场，先培训后输出，对参加培训的农民给予补贴，着力提高务工人员的基本技能。努力巩固已有的劳务基地，开辟新的劳务市场，加大劳动维权力度，提高劳务输出的组织化程度，实现劳务输出由数量型向效益型转变。力争全年有组织的劳务输出达到10万人，确保劳务输出稳定在40万人的规模。

　　加大对农业的支持和保护力度。依法加强耕地保护，确保占补平衡。毫不动摇地抓好“人均一亩基本农田”和“小工程大群体”的农田与水利基本建设，稳定粮食综合生产能力，确保粮食产量80万吨以上。按照“整村推进、工作到户、集中项目、捆绑资金”的要求，扎实抓好100个扶贫重点村建设，每村投入资金不少于100万元。易地扶贫搬迁6000人，实现5万人脱贫。认真落实兑现退耕还林和工程造林植竹补助奖励资金，抓好汉滨区粮食直补资金的兑现落实，不折不扣地执行支持农业、减负增收的各项政策措施。增强农村信用社支农服务的能力，加大财政对农业的投入。在中省决定取消我市8个国家贫困县区农业税的同时，由市统筹，全面取消10县区农业税，让全市农民同步减负，得以休养生息。

　　（二）深化国有企业改革，加快工业经济发展

　　深化国企改革。继续抽调精兵强将，实行一户企业一名领导挂帅、一个工作组包抓、一套改制方案的办法，年内基本完成国企改革任务。按照建立现代企业制度的要求，因企施策，采取兼并重组、股份制改造、依法拍卖、政策性破产等多种形式，使国有资本有序退出，职工得到妥善安置。在资产重组中努力实现资本扩张，在企业破产中尽可能破产不破业，减少职工下岗。正确处理政府、企业、职代会的关系，既要充分尊重职工的民主权利，又要维护企业的经营自主权，更要确保政府作为国有资本出资人的地位。正确处理改革与稳定的关系，把握好改革的时机与推进力度，确保改革顺利进行。

　　壮大特色工业。生物制药关键在于整合资源、打造龙头。积极引进战略合作伙伴，依托特色资源，做靓品牌，力促正大制药、北大制药、天宝药业、镇坪制药厂等GMP认证企业尽快实现跨越式发展。突出发展绿色食品加工业，提高产品档次，扩大宣传营销，首先把泸康酒、富硒茶、秦东魔芋等产品做大做强。加快水电能源基地建设，配套发展载能工业。依靠改制、重组和改造，创新机制，加强管理，降耗提效，振兴茧丝加工业。提高冶金矿产建材开发准入门槛，高度重视环境保护、劳动保护和节约资源，坚决淘汰污染严重、浪费资源的小矿小厂，支持集约经营，实现规模开发。以通用公司为龙头，支持行业兼并重组，密切与二汽的合作，培育壮大机械加工集团，增强市场竞争力。

　　加强技术改造。鼓励企业技术创新，采用新技术、新工艺、新设备，着力实施一批能显著提升企业竞争力的技术创新和提质扩能项目。年内完成正大制药公司葛根素系列产品生产线、北大制药公司股蓝泼尼松生产线、通用公司汽车制动器总成生产线、五联矿业公司扩产等技改项目。完成工业技改投入6亿元以上。

　　（三）坚持项目带动战略，努力扩大投资规模

　　扎实抓好重点项目建设。竭力提供良好的建设环境，支持国家铁路和高速公路在我市加快建设。突出抓好本市安排的44个重点项目，确保完成投资30亿元。公路交通建设，以贯通石泉—汉阴—紫阳—岚皋—镇坪大南环连接线、平镇二级路、300公里通乡油路等为重点，完成投资3.5亿元。推进安康中心城市建设，扎实抓好大桥路综合改造、滨江大道二期等一批重点工程，继续抓好县城及重点集镇建设，实现城镇建设投资8亿元。加大生态环境建设与保护的投入。江南污水处理厂竣工投运，开工建设市垃圾处理厂，加快汉江沿线县城集镇环保设施建设。完成造林50万亩，水保治理500平方公里。继续抓好瀛湖风景区工程造林，今年基本完成绿化任务。水电开发坚持规划领先，重视流域开发对生态环境的影响，兼顾各业利益，完成投资10.5亿元。

　　扩大招商引资。制定招商引资工作细则，建立招商引资目标责任制。把“龙舟节”真正办成对外开放、招商引资的平台。充分发挥在安投资商的作用，有重点地做好以商招商、以商引商的工作，采取上门招商、小分队招商等多种形式，主动走出去、请进来。精心策划包装一批好项目向外推介，扩大引资成果。争取项目签约、资金到位高于去年，完成招商投资10亿元以上。

　　盘活非经营性国有资产。优化配置非经营性国有资产，加速政府资产的合理流转，对地处安康城区的党政事业单位资产统一规划，整体置换，分步实施。加快行政中心、文化艺术中心、老干部活动中心建设。刺激政府在公共服务领域的消费，带动社会投资，拉动中心城市建设，促进经济增长。

　　（四）发展壮大旅游产业，着力培育消费市场

　　按照大旅游、大市场、大产业的思路，把旅游业真正培育成拉动消费的龙头和全市经济的主导产业。进一步突出生态旅游主题，把自然风光、消费城市、休闲旅游作为全市旅游业发展的基本定位，彰显特色，增强卖点。高起点抓好规划修编，严格按规划实施重点景区景点建设。培育以瀛湖品牌为代表的一批旅游精品，加大旅游产品开发力度。重视“红色旅游”资源的开发与保护。加强重点景区景点基础设施和精品线路建设，建成多功能星级观光游船，开辟瀛湖至紫阳水上旅游线路，增强景区景点的吸引力和游娱项目的参与性。整顿旅游市场，规范旅行社经营行为。认真办好“龙舟节”、“五一”、“十一”黄金周等节庆活动，主动到西安、陕北、关中、十堰、达州等地推介我市旅游产品和项目，促进客源互流，推进旅游投资与经营的合作。适应休闲旅游的市场需求，继续抓好地方风味小吃和旅游纪念品开发，推出茶艺品茗、地方戏曲、山歌民俗、药浴药膳等特色消费。突出抓好宾馆、饭店、“农家乐”住宿和餐饮条件的升级改造，提升服务档次。全年实现接待游客130万人次，综合收入4亿元。

　　培育住房市场，推动住房消费。加强城市规划和管理，完善市政基础设施，促进房地产业健康快速发展。认真执行经济适用住房政策，鼓励发展经济适用住房，适度发展中高档商品房，有计划地建设廉租房，彻底实现住房商品化。培育住房有形市场，积极拓展二手房和住房租赁市场，促进住房交易流转。认真实施《城市物业管理办法》，切实改善住房消费环境。通过住房消费带动装修装饰、家具家电等行业发展。

以促进农产品流通、提高农民购买力为重点，积极扩大农村消费。加快集贸市场建设，创造农产品进城入市的条件，鼓励经纪人、运销户经销农副产品，促进货畅其流，把农民增收真正转化为现实购买力。加强农村消费市场的监管，严厉打击制假售假、坑农害农行为，维护农民消费权益，努力扩大农村消费。

　　（五）进一步优化投资环境，放手发展非公有制经济

　　把改善投资环境作为生命线工程来抓。全面实行政府部门办事内容、办事程序、办事权限、办事时限、办事结果以及收费依据和标准“六公开”。整顿和规范市场经济秩序。建立客商评定投资环境机制，及时受理客商投诉，坚决遏制乱收费、乱罚款、乱摊派。从重从快打击侵害外来投资商财产人身权益的违法犯罪行为。热心解决外来客商子女就学等实际困难。

　　放手发展非公有制经济。鼓励非公有资本进入基础设施、公用事业及其他法律法规未禁止的行业和领域。支持非公有制企业参与国有企业的改组改制。鼓励非公有资本参与竞争政府投资项目。对非公有资本投资项目，政府有关职能部门尽力做好指导协调工作，并由审批制转为核准制或备案制。在税收、用地、用工等方面与其他经济成分一视同仁。完善企业登记并联许可办法。鼓励企业依法开展“联户联保”等形式的互助性融资，积极组建中小企业信贷担保机构，解决贷款难问题。重视发挥工商联及民间商会的作用，加强信息咨询和协调服务。引导和扶持非公有制企业开展技术创新和管理创新，不断提高自身素质。

　　（六）加快发展社会事业，推动社会全面进步

　　促进教育事业加快发展。完成安康学院、职业技术学院组建工作。巩固办好重点县级职教中心。加快发展高中教育，力争初中毕业生升入普通高中的比例达到38%以上，高中在校生达到4.5万人。高度重视基础教育，巩固“普九”成果，努力增加投入。紫阳县要在今年实现“普九”达标。加大学校危房的改造力度，对义务教育阶段的教师工资和学校经费实行财政全额预算，对各项政策性收费不得抵扣财政支出。

　　加强科技文化工作。重视多层次人才队伍建设，加强引智工作，扩大对外技术合作与交流。鼓励科技人员直接从事科技成果转化和生产经营，围绕主导产业抓好实用技术成果的推广普及。提升月河流域农业科技示范带建设水平。繁荣文化事业，发展文化产业，着力打造汉剧、民歌等汉水文化品牌。广泛开展全民健身活动，办好“安康市第十三届运动会”。加强信息化建设，推进电子政务，抓好广播电视“村村通”。

健全公共卫生防治体系。全面完成各县区疾控中心国债项目建设，完成40所乡镇卫生院新建和改扩建任务。改革疾控中心、妇幼保健、乡镇卫生院财政供给办法，确保公共卫生享受公共财政。切实抓好地方病、传染病、职业病防治和母婴保健。对主动接受艾滋病检测的人员，其检测费用由财政负担。高度重视食品、药品、饮水安全。深入开展爱国卫生运动，整治城市脏乱差，改善市容市貌。

　　巩固低生育水平。进一步夯实基层基础工作，强化人口计划生育工作目标责任管理，落实计划生育各项优惠政策。稳定低生育水平，控制出生人口性别比失调。

　　认真做好“十一五”规划的编制工作，充分吸收各方面的意见和建议，为未来安康的发展绘制新的蓝图。

　　三、努力建设以人为本的和谐安康

　　以人为本，构建和谐社会，是科学发展观的本质要求，集中反映了最广大人民群众的根本利益，是政府职能的体现和政府工作的追求。

　　（一）发挥财政职能，促进社会公平

　　调整财政支出结构。将政府职能真正转向对公共事务的管理与服务，将财政支出的重点转到维护公平分配、关顾弱势群体上来。集中财力加大对科教文卫、公共产品、社会保障和“三农”的投入。调整完善市对县区财政体制，明确市县区支出责任，确保干部工资和离退休干部生活费按时发放，离休干部医疗费按规定实报实销。加强对支出的监控，厉

行节约，反对浪费，降低行政成本。运用财税杠杆，加强对高收入行业和群体的税收征管，调节部门、行业之间的分配不公，加大对灾区、条件艰苦地区和财力过低地区的转移支付，促进社会公平。

　　完善社会保障体系。在巩固职工养老和医疗保险的基础上，足额预算城市低收入人口生活保障金，做到应保尽保，真正建立起标准统一、资金落实的城市低保制度。抓好下岗职工基本生活保障向失业保险并轨。全面启动建立农村居民最低生活保障制度。扎实抓好旬阳县和全市五保户农村合作医疗保险试点，力争通过三至五年的努力，使合作医疗保险覆盖全市农村，从根本上改善农民看病难问题。对符合政策的农村计划生育对象每人每年发放奖励扶助金600元，解决好计划生育家庭老有所养的问题。从今年起，对确认的39936名贫困家庭的适龄儿童，一律免收书费和杂费，给予寄校生活补贴。从建立和完善这些制度开始，让公共财政的阳光真正照耀到最需要党和政府提供帮助的弱势群体和困难群众。

　　（二）关心群众生活，维护群众利益

　　抓好就业再就业工作。政府、社会和求职者共同努力，拓宽就业空间。大力发展劳动密集型产业，鼓励三产服务业加快发展，充分吸纳就业。对下岗职工自主创业或安置下岗职工达到一定比例的企业，按规定实施税费减免。积极开发公益性岗位，专门安置下岗职工。充分发挥街道社区职业介绍和就业指导作用，积极发展社区服务和家政服务。做好下岗职工创业小额信贷担保工作。重视残疾人就业。引导大中专学生转变择业观念，坚持面向市场就业，积极为他们提供就业信息、档案管理、人事代理等服务。新增城镇就业9500人，登记失业率控制在4 %以内。

重视解决群众关心的具体困难。继续抓好边远村落便民桥建设，在原有投资标准的基础上追加300万元，再新建300座便民桥。完成安康城区背街小巷改造任务，着手解决群众如厕难、交通秩序差等反映强烈的问题。投资2500万元新修和改造35处人畜饮水工程，解决7万人饮水困难。投资1950万元，实施9.5万户防氟改造。政府安排专款，用于农村儿童计划免疫，确保接种率达到90%以上。

　　（三）强化基础工作，确保社会稳定

　　长治久安是人民的期盼，是构建和谐社会的重要目标。以人民群众的安全感为第一信号，从加强社会治安综合治理入手，加大“四五”普法力度，加强对未成年人的教育和保护，及时侦破大案要案，从重从快惩处严重违法犯罪行为，严厉打击黑恶势力、街痞村霸，确保一方平安。夯实安全生产责任，完善安全生产预案，强化安全生产基础工作，建立安全生产长效管理机制，坚决遏止重特大安全事故发生。健全灾害预警机制，扎实做好防灾减灾工作，确保人民群众生命财产安全。认真接待和办理群众来信来访，加大信访积案、要案办理力度，依法规范信访秩序，引导群众以理性合法的方式表达利益诉求。

　　维护稳定的基础在基层。基层稳定、民心稳定才能实现持久的稳定。加强基层民主政治建设，保障人民群众的知情权、参与权、建议权、监督权，增强民主管理的意识和能力。坚持工作重心下移，把维护稳定的着力点放到基层，把心操在基层，把不稳定因素化解在基层，努力营造稳定祥和的社会环境。

　　（四）自觉接受监督，加强政府建设

　　坚决维护《宪法》和法律权威，根据法律授予的权限和程序行使权力。自觉接受人民代表大会及其常委会的法律监督、工作监督和人民政协的民主监督。推行“阳光行政”，增强政府工作透明度，接受舆论监督和公众监督。加大从源头上预防和治理腐败的力度，纠正部门和行业不正之风，从严查处政府工作人员违法违纪案件，坚决刹住国家公职人员参与赌博。改进政风，转变作风，坚决反对官僚主义、形式主义，下力整治软、懒、散、浮，严明纪律，令行禁止，维护政令畅通。

　　构建和谐社会要求政府把职能转到经济调节、市场监管、社会管理、公共服务上来，努力建设学习型、服务型、法制型、落实型政府。政府工作人员要努力适应新形势新任务的要求，不断提高正确判断形势、民主科学决策的能力，提高遵循经济规律、充分发挥市场机制作用的能力，提高统筹兼顾、综合协调的能力，提高处理复杂问题、应对突发事件的能力，提高坚持以人为本、构建和谐社会的能力。

　　诚信建设是构建和谐社会的基石。政府诚信建设的水平是和谐安康的标志，必须维护公平，坚持正义，真正成为人民群众信任的政府。市长和政府组成人员的诚信修养代表着政府形象，必须公道正派，清风善政，真正成为人民群众放心的公仆。

　　各位代表！我们的事业是安康人民的事业，294万人民的希望就是我们的责任。让我们在邓小平理论和“三个代表”重要思想指引下，精诚团结，众志成城，以求真务实、百折不挠的精神，开创我市改革与发展的新局面，把安康人民的事业不断推向前进！
